# Experiential and Doctrinal Religious Knowledge Categorization in Parkinson's Disease: Behavioral and Brain Correlates

**DOI:** 10.3389/fnhum.2016.00113

**Published:** 2016-03-22

**Authors:** Edward J. Modestino, Partrick O'Toole, AnnaMarie Reinhofer

**Affiliations:** Department of Neurology, Boston University School of MedicineBoston, MA, USA

**Keywords:** doctrinal, experiential, Parkinson's disease, religious cognition, functional connectivity

## Abstract

Recent studies suggest changes in religious cognition in a subgroup of patients with Parkinson's disease (PD e.g., Butler et al., 2011). It is unclear whether this deficit extends to both doctrinal and experiential categorization forms of religious cognition. Kapogiannis et al. (2009b) dissociated experiential and doctrinal religious knowledge to different neural networks using fMRI. We examined Kapogiannis' dissociation against the background of PD side of onset (LOPD, ROPD), assessing performance both On- and Off-medication. In the behavioral portion of the study, we used a statement classification task in combination with scholar derived test sets for experiential and doctrinal religious knowledge categorization in conjunction with neuropsychological measures. In the neuroimaging portion of the study, we expanded on Kapogiannis' study by examining the same networks in PD. The behavioral data revealed that all groups rated (categorized) the scholar derived tests of experiential and doctrinal significantly differently than the scholars. All groups, including the scholars, classified more phrases as doctrinal than experiential. Religious cognition differed in the PD groups: those with PD Off-medication and LOPD Off-medication comprehended scholar defined experiential phrases with more difficulty, making them more likely to be classified as mixed or doctrinal. This was in contrast to the subjective frequency of classification of phrases as experiential paired with a cognitive decline in PD Off-medication; whereas PD On-medication showed a positive correlation with cognitive state and subjective doctrinal classification. For ROPD, cognitive state was associated with subjective experiential and doctrinal frequency of classification. With more intact intellect, there was a greater likelihood of classifying phrases subjectively as mixed, and the converse for experiential. Furthermore, religiosity negatively predicted subjective doctrinal frequency in LOPD, with the converse in ROPD. In fcMRI in PD, we found resting state functional intrinsic connectivity of reward networks associated with classification of statements using seeds in bilateral nucleus accumbens in PD. For experiential regressors, there was a negative correlation in bilateral frontal lobes paired with a positive correlation in left occipital visual areas (BAs 17, 18). For doctrinal regressors, there was a positive correlation in right BA 20.

## Introduction

Parkinson's disease (PD) is widely known as a degenerative motor disease that involves gradual loss of dopaminergic neurons in the striatum (Bell et al., 2014). However, increasing evidence has shown a variety of cognitive (Ventura et al., 2012; Maril et al., 2013) and psychiatric (Cubo et al., 2010) impairments associated with the progressive degeneration. Furthermore, recent studies have shown changes in religiosity and religious cognition associated with the development of PD. McNamara et al. (2006) demonstrated reduced religiosity in PD (*n* = 22) vs. controls (*n* = 20) using the Brief Multidimensional Measure of Religiousness/Spirituality (BMMRS; Fetzer Institute, 1999). Butler et al. (2011) was able to show that those with PD (*n* = 71) were significantly lower in religiosity on the BMMRS than controls (*n* = 75), with left-onset PD (LOPD) patients showing the greatest impairment with regard to religious cognition. Similarly, Giaquinto et al. (2011) in a study with 83 PD patients showed a significant difference between controls (*n* = 79) and LOPD (*n* = 31), with controls scoring higher across various subscales of religiosity using the Royal Free Interview (King et al., 1995).

Religious cognition has been modeled (Kapogiannis et al., 2009b) in terms of three psychological dimensions, namely, God's perceived involvement; God's perceived emotion and basic knowledge sources: the experiential and doctrinal. We focus on experiential vs. doctrinal knowledge categorization sources in this paper. Glock (1962) included the experiential as one of the five dimensions of his Model of Religious Commitment. He gave examples such as speaking in tongues, conversion experience, experiencing the Holy Spirit, and a religious feeling not necessarily associated with formal and public religious practice. Kaufman (1966) described the experiential as feeling one's place in relation to God, one's creator, after being thrust into a world outside one's control. He emphasized the finitude of human life and experiences of transcending such limitations. Himmerlfarb (1975) categorized experiential to include feeling the presence of God, trust and fear of God, and visions which may include miracles. Alston (1986) equated experiential with perception of evidence to confirm our beliefs; whereas Davidson (2003) characterized it as related to our personal relationship with a supernatural entity and our desire for related experiences, extending Glock's model. Whitehouse explained the experiential with “imagistic” modes of cognition and recognized that it involves episodic memory (Whitehouse, 2002). Gibson and Zahl (2012), called the experiential “heart knowledge,” stating it was “affect-laden” and based on personal beliefs in, and experiences of, God.

Various neuroimaging studies have investigated the experiential religious knowledge categorization. Hayward et al. (2011) used volumetric analysis of neuroanatomy with MRI to look for brain volume changes associated with the experiential. They found that there was significantly less atrophy of the left orbitofrontal cortex in those with who claimed to have a “life-changing religious or spiritual experience.” Kapogiannis et al. (2009a) also examined brain volume in relation to the experiential. Using voxel-based morphometry of neuroanatomical MRIs, they showed that experiencing a personal relationship with God corresponded with increased cortical volume of the right middle temporal gyrus extending toward the temporal pole. Those who claim to experience “fear of God's anger” had a decrease in cortical volume of the left precuneus and left orbitofrontal cortex; whereas positive experiences of God were associated with an increased cortical volume in these areas. Using functional MRI, Kapogiannis et al. (2009b) was the first to examine explicitly neural correlates along a spectrum ranging from experiential to doctrinal categorization. In the cognitive domain, experiential was associated with high-imagery and episodic memory retrieval. Brain correlates of experiential included bilateral occipital lobes, the left precuneus, left precentral gyrus, and the left inferior frontal gyrus. In the cognitive domain, doctrinal was associated with deriving meaning related to metaphors and use of semantic memory. Brain correlates of doctrinal included the right inferior temporal gyrus, right middle temporal gyrus, right inferior parietal/supramarginal gyrus, left cingulate, and the left superior temporal gyrus.

On a spectrum, the dichotomous pole to the experiential is the doctrinal. Whereas experiential knowledge is based upon concrete experiences, doctrinal knowledge is abstract and learned from religious texts and education. Glock (1962) also included the doctrinal within his model, specifically under the ideological dimension of religious belief. Himmerlfarb (1975) classified doctrinal as “belief in major tenets of faith.” Whitehouse (2002) characterized the doctrinal as highly structured and integrated with the learning of religious teachings. Additionally, he stated that rituals related to doctrine involved semantic memory and are used to create unity within a large society (Whitehouse and Lanman, 2014). Gibson and Zahl (2012) defined doctrinal as “head knowledge” in contrast to “heart knowledge” as experiential; it concerns what people think they should believe in relation to God based on religious teachings and tomes.

As it is common knowledge that motor symptoms in PD tend to emerge asymmetrically, contralateral to hemisphere of greatest brain degeneration (Riederer and Sian-Hülsmann, 2012), and changes in religiosity have been seen in PD relative to side of onset (Butler et al., 2011; Giaquinto et al., 2011), we decided to expand on the study of Kapogiannis et al. (2009b) to examine experiential and doctrinal religious knowledge categorization within PD. Although, there appears to be a deficit in general religious cognition in some patients with PD as mentioned above, it is not clear that these deficits extend to, or are specific to, doctrinal religious knowledge (and associated semantic memory and primarily right hemispheric brain regions as suggested by Kapogiannis et al., 2009b) or experiential religious knowledge (and associated episodic memory and primarily left hemispheric brain regions as suggested by Kapogiannis et al., 2009b); and if lateralized brain degeneration in PD is associated with deficits in either of these religious knowledge networks preferentially, e.g., LOPD showing a deficit in doctrinal religious knowledge due to greater right hemispheric brain degeneration, or ROPD showing a deficit in experiential religious knowledge due to greater left hemispheric brain degeneration. However, recent evidence has shown that those with PD have significantly lower measures on semantic and episodic memory than controls (Varrone et al., 2015). All of this is the foundation of this study.

Our first objective was to take the 68 phrases used by Kapogiannis et al. (2009b), which were touted as falling along a spectrum ranging from experiential to doctrinal religious knowledge categorization, to create objective test sets (experiential, doctrinal, and mixed—some combination of the first two categories) based on ratings from academic scholars of religious studies. Our next objective was to create a computerized task using these phrases to examine the way in which patients with PD [PD subgroups were side of onset (left-onset PD (LOPD) and right-onset PD (ROPD), with the added layer of On-medication and Off-medication, refraining from first daily dose of medication for PD)] subjectively classify/categorized these phrases (experiential, doctrinal, or mixed) in relation to the scholar derived test sets, each other and in relation to cognitive/neuropsychological measures. [It is important to acknowledge that we were interested in religious knowledge categorization along a spectrum ranging from experiential to doctrinal, independent of participants' religious affiliations (or lack thereof) and/or personal religious beliefs (or lack thereof). For a full explanation, please refer to Section Procedures: Behavioral Experiment for the instructions given during the task]. With these objectives, we had the following hypotheses. Hypothesis 1: Those with LOPD might have difficulty in classifying doctrinal as shown by significant differences from the scholars, but no difference in classifying experiential from scholars. Hypothesis 2: ROPD may have difficulty in classifying experiential, as shown by significant differences from scholars, but no difference in classifying doctrinal from scholars. Hypothesis 3: A more data driven approach will reveal where cognitive changes associated with LOPD, ROPD, or On- and Off-medication, may be associated with significant differences between subjective doctrinal and experiential classification frequency. [Note: This is subjective frequency of classification which is independent of the agreement with the scholar derived test sets of ratings].

Finally, with the neuroimaging portion of the study, our objective was to examine the resting state brain networks associated with experiential and doctrinal religious classification in PD using behavioral regressors (covariates) from each participant's own classification (outside of the scanner) of the phrases using a subgroup of those from the behavioral study. Thus, the neuroimaging component was directly expanding on the study of Kapogiannis et al. (2009b) by attempting to examine these very same networks in PD On-medication, which had never been done to our knowledge. Our hypotheses here were that an overlap with the networks found by Kapogiannis et al. (2009b) for experiential (Hypothesis 4) and doctrinal (Hypothesis 5) would be found within our data. For this component, we chose specific seeds to be used in the resting state fcMRI (functional connectivity magnetic resonance imaging) based on known degeneration of these dopaminergically innervated regions in PD, which we assume are intertwined with connectivity networks associated with the changes in religiosity seen in PD (Butler et al., 2011; Giaquinto et al., 2011). Changes in the dorsal and ventral striatum are well documented in PD with ventral striatal changes (i.e., the nucleus accumbens) most consistently associated with personality and behavioral changes in PD (McNamara, 2011; Carriere et al., 2014). Thus, we selected bilateral nucleus accumbens as regions of interest-seeds, to examine connectivity with other brain regions.

## Methods

### Participants: Behavioral experiment

In the behavioral experiment, the participants included patients (*n* = 35; 34 males, one female) diagnosed with idiopathic Parkinson's disease, by a board certified movement disorders specialist and Director of Movement Disorders clinics at the Boston VA who recruited patients for the study from the Veteran's Administration Health System in Boston, MA, USA. [Note: One male LOPD subject failed to do the task and was thus dropped from the analysis of phrase categorization. Thus, the final PD total was *n* = 34]. Based on our recruitment at the VA, the majority (*n* = 33) were veterans; whereas two participants were recruited from local area PD support groups. The PD group mean age was 68.34 within an age range of 42–89. The majority of PD patients were Caucasian (*n* = 32), two were African-American, and one was Asian-American. The majority were right handed (*n* = 28), four were left handed, and three were ambidextrous. All but two were high school graduates (*n* = 33), many had college degrees or a minimum of two years college education (*n* = 25) and some had postgraduate degrees or at least one year of post-baccalaureate education (*n* = 10). The mean amount of education among the PD patients was 15.01 years. Within the PD group (*n* = 34), there were 16 left-onset (including one male who failed to do the task, leaving 15 left-onset patients with usable data) and 19 right-motor onset patients. Five left-onset and 14 right-onset patients completed the task both On- and Off-medication; whereas others only completed either On- and Off-medication [total On-medication (*n* = 25): 8 LOPD, 17 ROPD; total Off-medication (*n* = 26): 11 LOPD, 15 ROPD]. The overall Hoehn and Yahr scale score (Hoehn and Yahr, 1967) mean was 2.54, within a range of 2:4, with the majority being 2 (*n* = 18), 3 (*n* = 12), and 4 (*n* = 3); two were unknown as they were recruited from a local PD support group. Thus, nearly 86% (85.71%, *n* = 30) were in stages 2–3. Length of PD duration had a mean across the group of 7.09 years within a range of 1.5–20 years.

### Participants: Neuroimaging

In the neuroimaging component of this study, 14 PD On-medication patients, a subset from the behavioral portion of the study, completed resting state functional connectivity MRI (rs-fcMRI). In the analysis of this fcMRI data, we used regressors derived from the participants' prior categorization in the behavioral task. The PD fMRI participants included: 14 males (mean age of 64.85, age range of 42–89); 13 were Caucasians and one Asian-American. They had 15.35 mean years of education within a range of 8–20. Ten were right handed, three left handed and one ambidextrous. The mean duration of PD was 6.96 years within a range of 2–18 years. The mean Hoehn and Yahr scale score of 2.5 within a range of 2–3. Ten were right-onset PD (ROPD) and 4 left-onset PD (LOPD).

This study (both behavioral and neuroimaging components) was approved by the Institutional Review Board (IRB) of VA Boston Healthcare System in Jamaica Plain, Boston, MA, USA. All participants completed an informed consent as specified and approved by said IRB. Participants interested in the MRI portion underwent an extensive screening to make sure they were eligible, free of MRI incompatible implants and claustrophobia, etc., as specified and approved by the IRB.

### Procedures: Behavioral experiment

We created a task in E-Prime (version 2.0; Psychology Software Tools^1^, Inc., Sharpsburg, PA, USA) using the 68 phrases from Kapogiannis et al. (2009b), which they showed could be easily rated/categorized along a continuum of experiential to doctrinal religious knowledge. This was a self-paced task with detailed instructions on a laptop (Toshiba Satellite L855 running Windows 8). Each of the 68 phrases needed to be rated, using a response button box, which also allowed the collection of response times. We ran 65 participants using an older response box (Serial Response Box, Model #200A, Psychological Software Tools, Inc., Sharpsburg, PA), which we subsequently replaced with a newer and more user-friendly one for the last 18 subjects (Response Pad Model RB-530, Cedrus Corporation, San Pedro, CA). [Please note that some of these participants included here are the same subject counted twice as they used both response boxes, as a patient with PD completed both On- and Off-medication conditions at different times. However, we wanted to be explicit with the number of sessions run on which response button box]. Subjective ratings for each phrase were made using a Likert Scale of 1:7, where “1” was purely experiential, “4” was an equal mixture of experiential and doctrinal, and “7” was purely doctrinal. Examples of classifying phrases were given using phrases that were not part of the test set of 68 phrases. The following are instructions that were given within the task:

“To do this rating, you choose how to categorize each statement not based on your own personal beliefs, but on how you think a person in general would come to believe these statements. Categorize these statements not by whether you agree or disagree with them, but by how you think people come to believe them.”

“Doctrinal concepts: those that are learned through texts and teachings; they tend to be intellectual, and abstract concepts. ‘The world was created in seven days.’ We consider this doctrinal because it was from the Bible, a religious text.”

“Experiential concepts: a person can experience it, it is emotional, and it has practical implications. ‘God’s presence is peaceful.' We consider this experiential because someone emotionally experiences it.”

“Both Doctrinal and Experiential concepts: ‘Everything happens for a reason.’ We consider this as a mixture of both. Doctrinal: It is a teaching about God's wills (an abstract idea). Experiential: People may feel meaning and purpose in their lives (a practical idea). In this case, you may choose somewhere between both Doctrinal and Experiential.”

“The examples given here are how we chose to classify the phrases given. You need to classify them yourself.”

All participants were given a battery of neuropsychological tests to assess possible comorbid dementia and cognitive impairment. These included the Mini-Mental State Examination (MMSE; Nazem et al., 2009), the Montreal Cognitive Assessment (MoCA; Nasreddine et al., 2005), the Wechsler Test of Adult Reading (WTAR; Holdnack, 2001), and the Matrix Reasoning test which is a subtest within the Wechsler Adult Intelligence Scale (WAIS; Wechsler, 2008) and the Stroop (Stroop, 1935). Additionally, participants were assessed for mood function using the Depression, Anxiety, and Stress Scale (DASS; Henry and Crawford, 2005). Furthermore, participants were given a take-home packet that included various inventories. To measure religiosity, we used the Brief Multidimensional Measure of Religiousness/Spirituality. The BMMRS was used as our primary assessment of participants' religiosity. Based on the factor analysis by Johnstone et al. (2009), the BMMRS has six dimensions. These include positive (Cronbach's α-reliability = 0.95), and negative spiritual experiences (α = 0.90), forgiveness (α = 0.81), religious practices (α = 0.72), and positive (α = 0.60) and negative congregational support (α = 0.52). The BMMRS also has a normalized total score of religiosity ranging from 0 to 86, with a cut-off of >43 for high religiosity, which was our primary use of the measure in this study. Furthermore, we used the Religious Commitment Inventory (RCI; Worthington et al., 2003). Personality traits were measured by use of the Big Five Mini-Marker (How Accurately Can You Describe Yourself?) which scores for the five subcomponents [O: openness, C: conscientiousness, E: extroversion, A: agreeableness, and N: neuroticism (emotional Stability) (Saucier, 1994)]. Finally, as our participants consisted of those with PD, which is often comorbid with REM sleep behavior disorder (RBD), we gave the REM Behavior Disorder Questionnaire—Hong Kong (RBDQ-HK; Shen et al., 2014).

All participants in the behavioral portion of the study were paid $10 an hour for their participation. Levodopa equivalency dosages (LED), to be used in statistical analyses, were calculated using standardized formulae to compare dosing levels across the variety of dopamine replacement therapies that our participants with PD were taking (Tomlinson et al., 2010). With regard to the On-medication vs. Off-medication testing, the order of the testing was counterbalanced. Some completed testing On-medication first, and then weeks later the Off-medication; whereas other completed this in the reverse order.

### Procedures: Pre-test and post-test task calibrations

**Figure A1:**
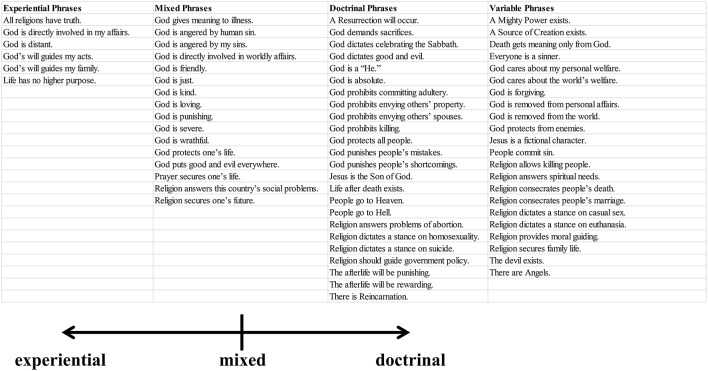
**These are the 68 phrases from Kapogiannis et al. (2009b) and their ratings along a spectrum from experiential to doctrinal by religious studies scholars**. Phrases in the mixed category were rated as equally experiential and doctrinal in nature by the religious studies scholars. Phrases in the variable category were inconsistently rated amongst scholars, and thus there was no consensus as to where they fit on the experiential to doctrinal spectrum.

In order to derive definitive test sets of phrases classified as experiential, doctrinal and mixed, we engaged 10 expert-scholars of religious studies (those with a graduate degree in religious studies or a related field such as theology) to rate the 68 phrases used in the behavioral study in a pre-test. For the scholars, we did not use the computerized version of the task. Instead, we emailed out the 68 phrases to have them rate them within a spreadsheet. Additionally, we included the same detailed explanation given within the computerized task. Furthermore, we included the Kapogiannis et al. (2009b) article. The scholars were instructed to email us back their ratings for each phrase within a spreadsheet. To this end, test sets of phrases were consistently classified by experts as experiential, mixed and doctrinal by ratings derived from the majority of scholars (using both the mean and mode of their ratings of categorization). Please refer to the Figure A1 in Appendix for specific phrases and the test sets.

In addition to the scholar derived test sets, we also performed a cluster analysis across all 68 phrases for the ratings from a cohort consisting of all PD participants. These analyses were done post-test to verify whether indeed there were distinct factors/clusters such as experiential and doctrinal present within the data.

### Behavioral data processing and statistical analysis

Behavioral data, including ratings of phrases and response times, were merged across several datasets using E-Merge and exported using E-DataAid (both tools within E-Prime version 2.0) into a tab-delimited text file. This was then imported into Excel (Microsoft Office 2010, Microsoft, Redmond, WA, USA) on a laptop (Dell Precision M6500 running Windows 7 Ultimate 64 bit) for data processing and analysis. Simple analysis of means and frequency were completed within Excel. Additionally, data was exported from Excel for hypothesis testing in IBM SPSS Statistics (version 22, IBM, Armonk, NY, USA) and SAS/STAT 9.4 for Windows (Copyright 2013).

For between groups (PD subgroups) simple comparisons of single measures, independent samples *t*-tests were employed. To compare PD subgroups with religious studies scholar defined test sets (experiential, mixed and doctrinal phrases, please refer to the Figure A1 in Appendix), one-sample *t*-tests using sample value (scholar rating) across each test set of phrases were contrasted with PD subgroups ratings. Furthermore, we used the scholar derived test sets to compare the patient's ratings and reaction times to these specific phrase test sets between LOPD and ROPD. To avoid making assumptions about an unknown distribution within small sample sizes of subgroups of participants, and the need to eliminate outliers and normalization of the data, non-parametric analyses were employed on reaction time data. (Note: All reaction times throughout the various analyses were in milliseconds.) For simple comparisons within groups of frequency or ratings per category we used the Wilcoxon Signed Ranks test, for between-groups comparisons of the frequency of ratings per category, we used the Mann-Whitney U test. In summary, independent-samples *t*-tests were used for ratings and frequency of ratings; whereas non-parametric analysis (Mann-Whitney U) for reasons explained were used for reaction times. All of these statistics were completed using IBM SPSS (version 22) on a laptop (Dell Precision M6500 running Windows 7 Ultimate 64 bit).

We employed multivariate mixed-effects linear regression analyses to test for associations between neuropsychological measures and the frequency of classification of experiential, doctrinal and mixed phrases. All models were adjusted for age, education, sex (gender), and handedness. We allowed for outcome-specific fixed effects and subject-specific and measure-specific random effects. These multivariate analyses are more realistic models of the outcomes than using independent regression models for each outcome. Since all information within each subject is utilized, we are able to provide more interpretable and consistent results than simpler statistical models. Moreover, the problem of multiple comparisons is removed when viewed from these models (Gelman et al., 2012). These multivariate models provide higher power for detecting small but clinically important differences compared to independent regression models for each outcome (Goldstein, 2010). Finally, exploratory cluster analysis was applied on the ratings of phrases from the PD cohort using the centroid method as applied with PROC CLUSTER. These analyses were performed using SAS (SAS/STAT 9.4 for Windows, Copyright 2013).

### Procedures: Neuroimaging

In our neuroimaging component of the study, behavioral data (from the main part of the study) on the frequency of classification of experiential and doctrinal phrases at the subject level was derived for use as regressors in resting state fcMRI analysis. Specifically, the ratio of the number of phrases classified as doctrinal or experiential divided by the total number of phrases (i.e., #/68) was used to create regressors for doctrinal and experiential classification frequency. All participants in neuroimaging component were compensated $50.

### MRI acquisition

MRI scans were obtained using a Siemens MAGNETOM Trio 3 Tesla MRI with a 12-channel head coil at the VA Boston Healthcare System in Jamaica Plain, Boston, MA, USA. For each participant, two T1-weighted neuroanatomical images were acquired using an MPRAGE (magnetization-prepared rapid acquired gradient echo) pulse sequence [specifications: 176 slices, TR: 2530 m.s., TE 3.32 m.s. (sometimes changed to 3.36, 3.37, or 3.39 depending on the recommendations of the console), FOV: 256 m.m., flip angle 7°, gaps skip 0.50, with a voxel size of 1^*^1^*^1 m.m.] for a total of 6:02 min for each full head scan. Additionally, three sets of T2^*^-weighted functional images of resting state (with participants instructed to keep their eyes open) were acquired for each participant using an Echo Planar image (EPI) sequence with a sensitivity to BOLD (blood oxygen level dependent) contrast (specifications: 38 slices per full head volume, TR: 3000 m.s., TE: 30 m.s., FOV: 192 m.m., flip 12 angle 90°, 3 m.m. gaps skip 0.8, voxel size 3^*^3^*^3.75 m.m.) for a total of 120 volumes and lasting approximately 6:08 min for each run.

### MRI data processing and analysis

MRI data processing and analysis was completed within FreeSurfer software (http://surfer.nmr.mgh.harvard.edu/). Both T1-weighted images were parcellated into cortical and segmented into subcortical gray matter, white matter, and CSF for each subject. These surface reconstructions were combined and then used for inter-subject alignment and for seed placement. Of the three BOLD scans obtained at the subject level, the two with the least movement were used. Time points with more than 0.4 mm of motion were removed from the analysis. These scans were motion corrected, smoothed on the surface at 15 m.m., and then concatenated. Next, the scans were band-pass filtered between 0.01 and 0.1 Hz., and the global signal, white matter signal, and ventricle CSF, and the motion time course were globally regressed out. Behavioral regressors (covariates) included frequency of classification of doctrinal and experiential phrases, which were paired with bilateral seeds from the nucleus accumbens for analysis of the resting state data within the general linear model (GLM) in FreeSurfer. The seeds were defined using the entire structure as described in Fischl et al. (2002). The time course for the seeds was averaged across the two hemispheres into a single time course for analysis. We then corrected for multiple comparisons using a Monte Carlo simulation within the GLM in FreeSurfer. Cluster locations were identified based on FreeSurfer parcellation and the FreeSurfer surface atlas aparc.annot (Desikan-Killiany Atlas). Brodmann areas were derived from MNI305, as were reported coordinates for the greatest correlation/significance within a cluster. All results were obtained using one group one covariate (OGOC) intercept/offset difference analysis where a group average of network maps from bilateral nucleus accumbens were derived and correlated with behavioral regressors (covariates) within the GLM in FreeSurfer.

## Results

### Comparisons within groups for the statement classification task

Overall, statements were categorically rated as doctrinal (>4 on a Likert scale) significantly more frequently than experiential across all groups. This was true for PD patients tested On- or Off-medication, and with left or right onset PD. Please refer to Table 1 for details.

**Table 1:** **These are the frequency of ratings within categories for the statement classification task**.

**Group**	***N***	**Doctrinal (Mean ± SEM)**	**Experiential (Mean ± SEM)**	**Doctrinal**			**Experiential**			**Statistical test**
				**25th**	**50th**	**75th**	**25th**	**50th**	**75th**	
PD On-meds	27	33.33 ± 2.06	15.37 ± 1.92	29	35	41	6	15	24	*Z* = 3.929
Left-Onset	9	35.78 ± 2.82	12.22 ± 3.97	29.5	36	43	4.5	7	20	*Z* = 4.001
Right-Onset	18	32.11 ± 2.75	16.94 ± 2.075	28.25	34	40	10.5	15.5	24.5	*Z* = 1.718
PD Off-meds	26	34.77 ± 2.56	14.08 ± 1.86	26.25	38	43	4	14.5	20	*Z* = 3.18
Left-Onset	11	38.82 ± 2.74	12.36 ± 3.19	32	40	43	4	10	20	*Z* = 2.845
Right-Onset	15	31.8 ± 3.83	15.33 ± 2.26	28.25	34	40	10.5	15.5	24.5	*Z* = 2.783

*Values here are for percentiles 25th, 50th, 75th are shown; all Z values are significant at p < 0.05*.

### Comparisons between groups for the statement classification task

As dopaminergic agonists bind to a subset of dopamine receptors in contrast to non-agonists such as levodopa, patients receiving dopaminergic agonists as part of their pharmacotherapy were compared with those that were not receiving these agonists on all behavioral measures. No significant differences were found. Furthermore, no other significant differences were found between groups with frequency of subjective classification.

### Comparisons within groups for the statement classifications in connection with other factors

We examined PD groups both On- and Off-medication with multiple neuropsychological measures to see if any covaried with the frequency of ratings per category. This was performed using multivariate mixed-effects linear regression analyses while controlling for factors such as age, education, sex (gender), and handedness. For those Off-medications in reference to experiential ratings frequency, there was a significant negative relationship with normalized MMSE scores, and a significant positive relationship with Stroop Word subcomponent scores. In contrast to the experiential results for those Off-medications, there was a significant positive relationship between normalized MMSE score and mixed rating frequency for those Off-medications. For those On-medications, both normalized MMSE, and Stroop word subcomponent scores showed a significant positive relationship with the frequency of doctrinal classifications. No other significant findings were found. Please refer to Table 2 for these results.

**Table 2:** **This table presents significant findings for the frequency of subjective ratings as experiential, mixed and doctrinal across PD (both On- and Off-medication) that covaried with other measures**.

	**Test**	**Estimate**	**Standard error**	**Significance**
**EXPERIENTIAL**				
Off	MMSE^*^	−0.6728	0.1583	*t*_(157)_ = −4.25, *p* < 0.0001
	Stroop_W^∧^	0.3825	0.1583	*t*_(157)_ = 2.42. *p* = 0.0168
**MIXED**				
Off	MMSE	0.2673	0.1081	*t*_(157)_ = 2.47, *p* = 0.0144
**DOCTRINAL**				
On	MMSE	0.4527	0.1526	*t*_(157)_ = 2.97, *p* = 0.0035
	Stroop_W	0.3091	0.1526	*t*_(157)_ = 2.03, *p* = 0.0445

*This analysis used multivariate mixed-effects linear regression with dependent variables of experiential, mixed and doctrinal religious classification divulging significant betas from other measures, adjusting for age, education sex (gender) and handedness. Positive t-values indicate a positive correlation with the dependent variable in question. Negative t-values indicate a negative correlation with the dependent variable in question*.

* MMSE, Mini Mental Status Exam;

∧ *Stroop_W, Stroop Word subcomponent*.

We continued the analysis using this same multivariate mixed-effects linear regression to examine any covariation of neuropsychological measures with frequency of classification of ratings across PD. Again, this was performed while controlling for factors such as age, education, sex (gender), and handedness. For the ROPD group, there was a significant finding for the MMSE in the negative direction, and a borderline significant finding in the negative direction for WTAR for experiential classification frequency. These same measures for ROPD reverse in direction, to the positive, and significantly so for the mixed classification frequency. For the doctrinal classification frequency, there were some interesting findings between LOPD and ROPD. LOPD significantly covaried in a negative direction with doctrinal frequency on Dtotal (from the BMMRS). For ROPD, the Dtotal was in the positive direction with doctrinal frequency, as was the RCI total. Please refer to Table 3 for these results.

**Table 3:** **This table presents significant findings for the frequency of subjective ratings as experiential, mixed and doctrinal across PD [ROPD (right-onset PD) and LOPD (left-onset PD)] that covaried with other measures**.

	**Test**	**Estimate**	**Standard error**	**Significance**
**EXPERIENTIAL**				
ROPD	MMSE	−0.5747	0.1224	*t*_(368)_ = −4.7, *p* < 0.0001
	WTAR	−0.2447	0.1245	*t*_(368)_ = −1.97, *p* = 0.05^*^
**MIXED**				
ROPD	MMSE	0.2491	0.08579	*t*_(368)_ = 2.9, *p* = 0.0039
	WTAR	0.2191	0.08677	*t*_(368)_ = 2.52, *p* = 0.012
**DOCTRINAL**				
LOPD	Dtotal	−1.1149	0.3473	*t*_(368)_ = −3.21, *p* = 0.0014
ROPD	Dtotal	0.2542	0.1313	*t*_(368)_ = 1.94, *p* = 0.0537^*^
	RCI	0.3038	0.1488	*t*_(368)_ = 2.04, *p* = 0.0419

*This analysis used multivariate mixed-effects linear regression with dependent variables of experiential, mixed and doctrinal religious classification divulging significant betas from other measures, adjusting for age, education sex (gender) and handedness. Positive t-values indicate a positive correlation with the dependent variable in question. Negative t-values indicate a negative correlation with the dependent variable in question*.

* *borderline significant*.

*DTotal, Brief Multidimensional Measurement of Religion/Spirituality total; MMSE, Mini Mental State Examination; RCI, Religious Commitment Inventory-10 total score; WTAR, Wechsler Test of Adult Reading standardized scores*.

### Religious studies scholar test set ratings vs. PD

Of the 68 phrases (from Kapogiannis et al., 2009b), the religious studies scholars rated six consistently as experiential, 16 as mixed (a combination of experiential and doctrinal), and 24 consistently as doctrinal. These were used as test sets. The remaining 22 phrases were rated inconsistently across all scholars, and thus were not used in the analysis for comparison with other groups. Please refer to the Figure A1 in Appendix for the specific phrases and test sets. All subgroups (LOPD and ROPD both On- and Off-medication) classified doctrinal and experiential phrases significantly differently than the religious studies scholars. For the scholar defined mixed experiential/doctrinal category, only the LOPD Off-medication group was significantly differently than scholars, with ratings significantly closer to doctrinal; whereas all other groups were not significantly differently than scholars. Please refer to Table 4 for details on these statistics. Finally, using the phrase test sets defined as experiential, mixed, and doctrinal by the scholars, we examined these test sets for differences in ratings and reaction times between LOPD and ROPD On-medication. This revealed nothing of significance.

**Table 4:** **One-sample *t*-tests contrasting religious studies scholars derived test sets for experiential, mixed (a combination of experiential and doctrinal) and doctrinal categorical religious phrase classification with PD subgroups**.

	**Experiential ratings**	**Mixed ratings**	**Doctrinal ratings**
LOPD Off vs. Scholars	*t*_(10)_ = 10.569; *p* < 0.001^*^	*t*_(10)_ = 2.980; *p* = 0.014^*^	*t*_(10)_ = −5.538; *p* < 0.001^*^
LOPD On vs. Scholars	*t*_(7)_ = 8.530; *p* < 0.001^*^	*t*_(7)_ = 1.868; *p* = 0.104	*t*_(7)_ = −4.335; *p* = 0.003^*^
ROPD Off vs. Scholars	*t*_(14)_ = 14.194; *p* < 0.001^*^	*t*_(14)_ = 2.137; *p* = 0.051	*t*_(14)_ = −7.631; *p* < 0.001^*^
ROPD On vs. Scholars	*t*_(16)_ = 11.158; *p* < 0.001^*^	*t*_(16)_ = 1.396; *p* = 0.182	*t*_(16)_ = −7.918; *p* < 0.001^*^

*Off, Off-medication; On, On-medication*,

* *significant*.

### Cluster analysis of task phrases

Previous factor analysis of ratings using a much larger dataset for this same task of categorizing the 68 phrases resulted in some 42 factors. The large number of factors resulted in an uninterpretable dimensional reduction. Thus, it was decided to use a cluster analysis of ratings on this PD cohort data. The cluster analysis divulged four clusters: one associated with doctrinal, two associated with mixed experiential/doctrinal and one associated with experiential religious knowledge classification. There were phrases as classified by the scholars that overlapped with these clusters: 15/24 phrases (62.5%) classified as doctrinal by the scholars were shown as doctrinal in the cluster analysis; 3/6 phrases (50%) classified as experiential by the scholars were shown as experiential in the cluster analysis; and 13/16 phrases (81.25%) classified as mixed by the scholars were shown as mixed in the cluster analysis. Thus, there was an agreement that was nearly 64.58% mean of agreement for these phrases between the scholars and the cluster analysis from the PD cohort.

### rs-fcMRI results

**Figure 1:**
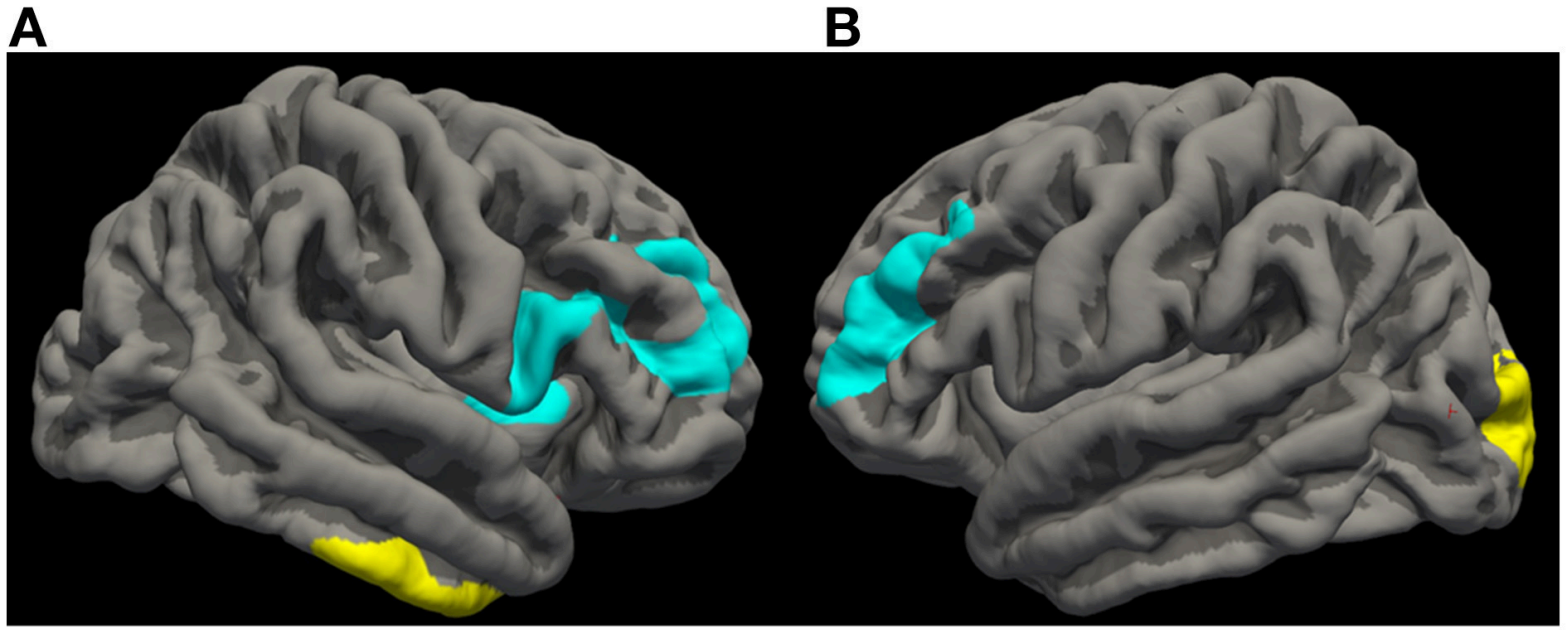
**Neuroimaging: Experiential and doctrinal resting-state functional connectivity networks associated with bilateral nucleus accumbens seeds in patients with Parkinson's disease (*n* = 14; 10 ROPD, 4 LOPD) On-medication**. On the left **(A)** is a composite group image of the right hemisphere including a blue cluster in the frontal lobe [with a peak in the pars opercularis of the inferior frontal gyrus (MNI305: 39.2, 9.2, 11.5) within Brodmann areas (BAs) 10, 46, 9, and 44, cluster size: 3712.54 mm^2^, *p* = 0.0001] of negative correlation associated with *experiential* religious classification and a yellow cluster in the temporal lobe [with a peak in the parahippocampal gyrus (MNI305: 31.4, −23.3, −24.9) within BA 20, cluster size:1725.59 mm^2^, *p* = 0.0032] of positive correlation associated with *doctrinal* religious classification. On the right **(B)** is a group image of the left hemisphere showing a blue cluster in the frontal lobe [with a peak in the rostral middle frontal region (MNI305: −36.5, 52.8, 0.9) within BAs 46 and 9, cluster size 2245.68 mm^2^, *p* = 0.0007] of negative correlation associated with *experiential* religious classification and a yellow cluster in the occipital lobe [with a peak in the lateral occipital lobe (MNI305: −20.4, −91.2, 14.2) within BAs 17 and 18, cluster size: 1437.22 mm^2^, *p* = 0.0184] of positive correlation also associated with *experiential* religious classification. All results were obtained using one group one covariate intercept/offset difference analysis within the GLM in FreeSurfer.

The one group one covariate (OGOC) results of patients with PD (*n* = 14) On-medication, using regresssors of the doctrinal frequency classification ratio at the subject level and bilateral nucleus accumbens seeds, showed a significant cluster of positive correlation in right temporal lobe [with a peak in the parahippocampal gyrus (MNI305: 31.4, −23.3, −24.9) within BA 20, cluster size 1725.59 mm^2^, *p* = 0.0032 (Figure 1A)].

The OGOC results of patients with PD (*n* = 14) On-medication, using regressors of the experiential frequency classification ratio at the subject level and bilateral nucleus accumbens seeds, showed three significant clusters. Two of these clusters were of negative correlation in the frontal lobes, one in the left [with a peak in the rostral middle frontal region (MNI305: −36.5, 52.8, 0.9) within BAs 46 and 9, cluster size: 2245.68 mm^2^, *p* = 0.0007 (Figure 1B)] and the other in the right frontal lobe [with a peak in the pars opercularis of the inferior frontal gyrus (MNI305: 39.2, 9.2, 11.5) within BAs 10, 46, 9, and 44, cluster size: 3712.54 mm^2^, *p* = 0.0001 (Figure 1A)]. Additionally, there was a cluster of significant positive correlation in the left occipital lobe [with a peak in the lateral occipital lobe (MNI305: −20.4, −91.2, 14.2) within BAs 17 and 18, cluster size 1437.22 mm^2^, *p* = 0.0184 (Figure 1B)].

## Discussion

### Behavioral findings

Across all PD groups (LOPD and ROPD both On- and Off-medication, and combined On- and Off-medication groups) phrases were repeatedly classified as doctrinal twice as often as experiential. This difference might be due to the simple fact that most anything related to religion for those who were not scholars of religious studies is often considered to be doctrine rather than a description of an experience. Furthermore, experiential religious classification may be a difficult concept for most people given that they may need to reflect on their own religious experiences to compare their experiences with that described in the stimulus phrase in order to verify that the two are congruent (Glock, 1962; Davidson, 2003; Gibson and Zahl, 2012). Importantly, this difference seen in all groups supports the claim that cognitive systems treat these two categories as distinct forms of religious knowledge.

With regard to our initial hypotheses, we did see evidence of changes in LOPD with regard to doctrinal classification, but not as predicted. Our hypothesis about ROPD having difficulty in classifying experiential phrases was not found specific to ROPD, since all groups, PD On-medication and ROPD On-medication, all showed significantly less classifications as experiential than doctrinal. Thus, it appears that everyone has difficulty with classifying the experiential, not just ROPD.

In the multivariate linear regression models, we were able to directly control for age, gender, education and handedness while examining all neuropsychological measures in relation to experiential, mixed and doctrinal classifications. When examining the result between PD On- and Off-medication groups, we discovered that those in the Off-medication group had an increase in experiential classifications paired with a negative relationship to MMSE score. Thus, with a general cognitive decline, those Off-medication would classify phrases more often as experiential. What is interesting about this is that this increase in experiential classification was also associated with the increase in the Stroop word score, which is a measure of processing speed. These results appear to be in contradiction with one another, as with a cognitive decline, there is usually an increase in processing speed. So, with greater processing speed, and a trend toward cognitive decline, those with PD Off-medication classified more phrases as experiential. Additionally, when these results are contrasted with the PD Off-medication group results for the mixed classification, there was a positive relationship with MMSE score, suggesting that with an increase in general cognition there was an increase in classifying phrases as mixed. For the PD On-medication group, both MMSE and Stroop word score were associated with increased classification of phrases as doctrinal. This suggests that with greater general cognitive function and faster processing speed, those PD On-medication classified more phrases as doctrinal.

In continuing with the multivariate regression analysis, still controlling for age, gender, education and handedness, we decided to test side of PD onset in relation to phrase classification and neuropsychological measures. For the ROPD group, there was an interesting dissociation between experiential and mixed phrase classifications. For the experiential classifications, the ROPD group revealed a negative relationship with MMSE score and WTAR (a verbal based premorbid intelligence test). So, ROPD with lower general cognition were more likely to classify phrases as experiential. This changed with regard to the mixed categorization where ROPD show a positive relationship to MMSE and WTAR. This suggests that for those with ROPD and greater cognitive skills, they are more likely to classify phrases as mixed. Finally, for the doctrinal classification, there was an interesting dissociation between LOPD and ROPD. For the LOPD, their Dtotal (BMMRS measure of religiosity) was in a negative relationship with classifying phrases as doctrinal. So, in essence, those who were less religious were more likely to classify phrases as doctrinal. However, for the ROPD, religiosity (Dtotal) and religious commitment (RCI) scores were positively associated with classifying phrases as doctrinal. The more religious and committed to religion they were, the more likely they were to classify phrases as doctrinal.

With regard to religiosity, we did not find a significant difference between LOPD and ROPD, as found in previous studies (Butler et al., 2011; Giaquinto et al., 2011). However, as the BMMRS was completed at home after the main testing, and subsequently mailed to return to us, many participants did not complete it. Furthermore, some completed it incorrectly or incompletely, which thus could not be scored. Therefore, the lack of difference between PD groups for religiosity may be due to the fact that only 54% of those with PD completed the BMMRS. However, there was an obvious trend as seen with LOPD having a lower mean score of religiosity ($\bar{X}=$ 27.5750) than that of ROPD ($\bar{X}$ = 39.1351). This is consistent with previous studies showing that those with LOPD have significantly lower scores of religiosity (Butler et al., 2011; Giaquinto et al., 2011). The possibility exists that the differences seen in previous studies were not seen in this study due to the low sample size of completed BMMRS measurements that were obtained from the PD group.

### Scholar derived test set findings

In the analysis which focused on using the religious studies scholars' derived test sets of the phrases, it appears that there were significant differences in comparison to non-scholars. Specifically, phrases that were consistently rated by scholars as experiential and doctrinal were consistently rated significantly differently than scholars by patients with PD. For the scholar-defined test set of experiential phrases, the PD groups consistently rated these phrases as mixed or even doctrinal. For the scholar-defined doctrinal test set of phrases, PD groups consistently defined these phrases as mixed and some doctrinal. Finally, for the scholar-defined mixed experiential/doctrinal test set of phrases, only the LOPD Off-medication were significantly differently than scholars, rating these phrases more often as doctrinal; whereas all other groups were not significantly differently than the scholars.

Although there are significant differences between PD subgroups from the objective test sets derived from the religious studies scholars for ratings of doctrinal and experiential classifications, when the PD cohort is taken as one group, cluster analysis has shown that there is a notable overlap (nearly 65%) in experiential, mixed and doctrinal classifications with that of the scholars.

### Neuroimaging findings

The fcMRI results of experiential and doctrinal religious knowledge revealed interesting findings with respect brain correlates of these two forms of religious cognition. Because LOPD and ROPD samples were so small, when contrasted there were no significant differences between them. It was then decided to combine the groups and see what was shared between them using behavioral regressors. For doctrinal regressors, paired with seeds in the bilateral nucleus accumbens (reward network), a significant positive correlation emerged within the right inferior temporal region in the parahippocampal gyrus (BA 20) in the intrinsic connectivity of resting state reward network. This right inferior temporal gyrus (BA20) has been associated with comprehension of metaphorical meaning (Ahrens et al., 2007) and concept abstractness (Cunningham et al., 2004). Interestingly, this temporal lobe region was reported to be associated with doctrinal religious knowledge in the Kapogiannis et al. (2009b) study. Kapogiannis et al. suggested that a doctrinal brain network was associated with deriving meaning related to metaphors and use of semantic memory—functions previously associated with BA 20. However, as this was not a task-based fMRI experiment, we must be cautious with our interpretation. We can state that the resting state reward network was positively associated with intrinsic connectivity in right BA 20 when paired with regressors for subjective classification of doctrinal religious knowledge. This same area is consistent with Kapogiannis' results of doctrinal religious knowledge (Kapogiannis et al., 2009b).

For experiential regressors, we discovered a bilateral negative correlation (left BAs 46 and 9, right BAs 46, 9, 10, and 44) in the prefrontal cortex linked with bilateral nucleus accumbens seeds in intrinsic connectivity of resting state reward network. This suggests that frontal areas associated with executive function are somehow negatively correlated (disengaged) with the intrinsic connectivity in the resting state reward network when paired with experiential religious knowledge categorization regressors. Furthermore, visual areas in the left hemisphere (BAs 17 and 18) showed a positive correlation with bilateral nucleus accumbens intrinsic connectivity with experiential regressors. The bilateral dorsal lateral prefrontal cortex (DLPFC: BAs 46 and 9) and the anterior prefrontal cortex (BA 10) have been associated with moral judgment (Moll et al., 2001); whereas the left pars opercularis (BA 44) is known as Broca's area and is involved in semantic processing (Goucha and Friederici, 2015). As these brain areas appear to be associated semantic processing and morality, and as they are negatively correlated here with regressors of experiential religious knowledge, we speculate that this may suggest a disengagement from some form of semantic processing (e.g., interpreting word abstractness; Roll et al., 2012) and possibly moral judgment. BA 17 (the primary visual cortex) and BA 18 (the secondary visual cortex) are associated with early visual processing (Miki et al., 2001). These visual areas were also seen by Kapogiannis et al. for experiential classification and were attributed to part of a neural circuit associated with high-visual imagery (Kapogiannis et al., 2009b). However, as this was not a task-based fMRI experiment, we must be cautious with the interpretation of this data. We can state that the resting state intrinsic connectivity of a reward network when paired with regressors for subjective classification of experiential religious knowledge classification, was negatively correlated with bilateral, frontal regions known to be involved with executive functions such as moral judgment and a left frontal region involved with semantic processing; and positively correlated with early visual areas. Again, these same early visual areas were seen to be associated with experiential religious knowledge by Kapogiannis et al. (2009b).

Despite the limitation of the sample size (*n* = 14) of patients with PD, our results revealed brain areas consistent with Kapogiannis' doctrinal and experiential religious knowledge categorization networks in neurotypicals. This was also consistent with our initial hypotheses for neuroimaging component of this study. However, the small sample size is of concern with regard to power. A power calculation is not relevant, as that would need to be completed prior to commencing an fMRI study (Mumford, 2012). *Post-hoc* power analyses done in an attempt to justify the power of the significant results of a completed fMRI study are pointless and potentially deceptive. In such a case, one can never be truly sure if results from such a study originated from the null distribution or another distribution (Hoenig and Heisey, 2001). Therefore, replication is the only way to authenticate these results. As increasing the sample size (n) will increase the power (Mumford, 2012), future replications should use considerably more participants. Based on a literature search of rs-fcMRI using behavioral regressors obtained outside the scanner, groups of participants ranging from *n* = 22 (Redcay et al., 2013) to *n* = 237 (Duchek et al., 2013) and even as high as *n* = 510 participants (Brier et al., 2012) have been published.

Differences between our results and those of Kapogiannis et al. (2009b) may be due to our theoretically motivated use of the bilateral nucleus accumbens as seeds in our analyses, and/or our use of behavioral regressors with resting state data rather than a task-based fMRI experiment. Future research with larger cohorts is needed to confirm these results, perhaps contrasting PD and neurotypicals, and to divulge any differences that may exist between LOPD and ROPD in relation to doctrinal and experiential categorization with fcMRI. Due to our limited sample size, we were not able to find significant differences between LOPD and ROPD between doctrinal and experiential classifications with fMRI. Furthermore, the intersection of semantic processing and moral judgment and their disengagement within a brain reward brain network, paired with engagement of early visual processing areas, all associated with experiential religious knowledge, needs to be elucidated in future research.

## Conclusion

In conclusion, with regard to our first two hypotheses, we found results far different than we theorized. LOPD and ROPD groups both classified doctrinal and experiential phrase test sets derived from the scholars significantly differently from the scholars. We were not able to see dissociation between LOPD and ROPD for doctrinal and experiential respectively using scholar derived test sets. However, it should be noted that all but one group, LOPD Off-medication, scored outside of significance for the mixed text set, rating mixed phrases. This is evidence that for the mixed test set, most groups were indeed in agreement with the scholars. As for LOPD Off-medication, they rated phrases from the mixed test set closer to doctrinal. Furthermore, there was a trend in LOPD to score lower on religiosity on the BMMRS than ROPD, which might have passed significance if the return rate on of the completed take-home packets (which included the BMMRS) had been greater than 54%. Additionally, it should be noted that despite the significant differences seen at the subgroup levels with the test sets, cluster analysis did reveal a considerable overlap between the PD-cohort when taken as a whole and the scholar derived test sets. Hypothesis 3 did indeed reveal differences in neuropsychological measures associated with frequency of subjective classifications within groups. For PD Off-medication, a general cognitive decline was associated with more experiential classifications; whereas a more intact cognitive function was associated with classifying things more frequently as mixed (which is moving closer to doctrinal) on the spectrum. So, when taken in conjunction with the scholar derived test sets comparisons, PD Off-medication rated scholar derived experiential phrases as closer to doctrinal, but for their subjective ratings of frequency, general cognitive state determined their subjective frequency of rating experiential or mixed. In continuing with Hypotheses 3, PD On-medication showed a positive correlation of general cognition and processing speed with classification frequency of doctrinal. For ROPD, there was a negative relationship between general cognition and premorbid intelligence with experiential frequency; whereas this relationship with cognition flipped to a positive one when moving toward the mixed category (closer to doctrinal) on the spectrum. For LOPD, religiosity negatively predicted doctrinal frequency; whereas for ROPD, religiosity and religious commitment positively predicted doctrinal frequency. Finally, with regard to Hypotheses 4 and 5, there was indeed an overlap with Kapogiannis et al. (2009b) for both experiential and doctrinal knowledge, suggesting two distinct intrinsic brain networks associated with them.

## Author contributions

All of the authors contributed equally in all aspects of the publication. EM was involved in the conception and design, acquisition, analysis, and interpretation of the results, drafting the work and revising it critically, final approval and agreement of being accountable for all aspects of the study. PO and AR were involved with interpretation of the data, revising the manuscript for intellectual content, final approval of the manuscript, and agreement to be accountable for all aspects of the study.

### Conflict of interest statement

The authors declare that the research was conducted in the absence of any commercial or financial relationships that could be construed as a potential conflict of interest.

## Footnotes

^1^Psychology Software Tools. (2014). *E-prime (Version 2) [Computer software]*. Available online at: http://www.pstnet.com/eprime.cfm.
